# Tribological and Antibacterial Properties of Polyetheretherketone Composites with Black Phosphorus Nanosheets

**DOI:** 10.3390/polym14061242

**Published:** 2022-03-19

**Authors:** Xuhui Sun, Chengcheng Yu, Lin Zhang, Jingcao Cao, Emrullah Hakan Kaleli, Guoxin Xie

**Affiliations:** 1 State Key Laboratory of Tribology, Department of Mechanical Engineering, Tsinghua University, Beijing 100084, China; sun-xh20@mails.tsinghua.edu.cn (X.S.); cjc2021@mail.tsinghua.edu.cn (J.C.); 2 Jihua Laboratory, Foshan 528200, China; yccycc9999@163.com; 3 Faculty of Mechanical Engineering, Automotive Division, Yildiz Technical University, Besiktas, Yildiz, 34349 Istanbul, Turkey; kaleli@yildiz.edu.tr

**Keywords:** black phosphorus, polyetheretherketone, lubrication properties, antibacterial properties

## Abstract

Over the past few decades, polyetheretherketone (PEEK) artificial bone joint materials faced problems of poor wear resistance and easy infection, which are not suitable for the growing demand of bone joints. The tribological behavior and wear mechanism of polyetheretherketone (PEEK)/polytetrafluoroethylene (PTFE) with black phosphorus (BP) nanosheets have been investigated under dry sliding friction. Compared with pure PEEK, the COF of PEEK/10 wt% PTFE/0.5 wt% BP was reduced by about 73% (from 0.369 to 0.097) and the wear rate decreased by approximately 95% (from 1.0 × 10^−4^ mm^3^/(N m) to 5.1 × 10^−6^ mm^3^/(N m)) owing to the lubrication of the BP transfer film. Moreover, BP can endow the PEEK composites with excellent biological wettability and antibacterial properties. The antibacterial rate of PEEK/PTFE/BP was assessed to be over 99.9%, which might help to solve the problem of PEEK implant inflammation. After comprehensive evaluation in this research, 0.5 wt% BP nanosheet-filled PEEK/PTFE material displayed the optimum lubrication and antibacterial properties, and thus could be considered as a potential candidate for its application in biomedical materials.

## 1. Introduction

For decades, the demand for artificial joints has been growing dramatically [1,2,3], while the research and development of new artificial bone joints has attracted extensive attention [4,5,6]. However, secondary injury after implantation, which is mainly caused by postoperative infection and wear of materials [7], is a key issue.

Polyetheretherketone (PEEK), a thermoplastic material with outstanding comprehensive properties, has been widely used in the manufacturing of aerospace items, electronic information products, automobile manufacturing, pharmaceutical and medical devices, etc., owing to its excellent biocompatibility, self-lubricating properties, chemical resistance, and good formability [8,9,10]. Since PEEK was first introduced into orthopedic joints in 1987 [11], it has been extensively used in the manufacture of artificial bone joints [12,13] and has been recognized by many medical device manufacturers and orthopedic surgeons owing to its biomechanical properties similar to those of human bones [14,15,16]. Even though its bioinertness remains a limitation for bone graft applications [12,17], PEEK implant modification by adding zirconia [18,19,20], cobalt alloy [21], titanium alloy [22], and so on improves the mechanical properties, biological activity, treatment of postoperative infection, and inflammation regulation of bone graft. Although PEEK possesses high raw material cost and high molding energy consumption [23], it exhibits the advantages of high strength, high temperature resistance, and chemical corrosion resistance [24], which are difficult to be matched by other polymers. PEEK can also be applied to manufacturing high value-added products [25,26].

Polytetrafluoroethylene (PTFE), an excellent solid lubricating material [27,28,29], has good self-lubricating performance when combined with PEEK [13,30,31]. Lin et al. [30] reported that the tribological characteristics of PEEK were enhanced by using a PTFE composite as a sacrificial tribofilm-generating part in a dual pin-on-disk tribometer. Haidar et al. [31] reported that the physical nature of transfer film adhesion by PEEK/PTFE could increase its wear tolerance to changes in environmental moisture.

**Figure 1:**
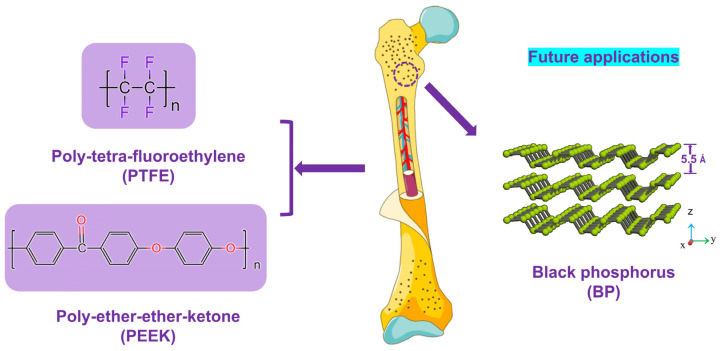
The schematic diagram and future applications of the PEEK/PTFE/BP artificial bone joint.

Black phosphorus (BP), a novel graphene-like two-dimensional material, has been successfully applied in the solid lubrication technology field [32,33] because of its unique fold structure and interlayer interaction by the van der Waals force [34,35,36,37]. Moreover, BP has broad prospects in biomedicine in virtue of the effect of bacteriostatic/bactericidal effects without cytotoxicity [38,39,40,41]. However, research results of the dual functions of the tribological and antibacterial properties mainly focused on the modification of alloy and ceramic materials [42,43,44,45], and only a few studies concentrated on the evaluation of the dual functions of PEEK and other polymers [7,46]. The main objective of this work is to develop a new type of PEEK composite material formulation with double functions of wear-resistance and antibacterial properties and dedicated to providing a new reference for solving the problem of secondary injury of artificial bone joints (see Figure 1). Therefore, the PEEK/PTFE/BP composite is evaluated from tribological and antibacterial aspects in this work.

## 2. Materials and Methods

### 2.1. Materials

The PEEK purchased from Victrex (450P), Lancashire, UK was used as the matrix, and the average particle diameter was 15 μm. The PTFE with a mean particle diameter of 12 μm was supplied by DuPont (MP1300), Wilmington, DE, USA. Red phosphorus (RP) powder (Aladdin, Shanghai, China, AR > 98.5%) with an average grain size of 15 μm was used as the raw material to prepare the BP nanosheets. The materials used in the antibacterial experiment were LB broth medium (Item No: A507002, Sangon, Shanghai, China), agar powder (Item No: A505255-0250, Sangon, Shanghai, China), *Staphylococcus aureus* (control No: ATCC29213, CGMCC), and phosphate buffered saline (Hopebio, Qingdao, China).

### 2.2. Preparation of Black Phosphorus Nanosheets

BP nanosheets were prepared using the high-energy ball-milling technique with RP as the raw material by using a planetary ball mill (Pulverisette 7, FRITSCH, Germany) at a speed of 800 rpm for 36 h. The ball-milling process was carried out alternately in the order of 25 min milling and 5 min suspension. The weight ratio of ball-to-powder was 20:1. Stainless steel balls with diameters of 4.5 mm, 6.5 mm, and 10.0 mm were employed as the ball-milling medium, and the weight ratio of the ball grinding medium was 1:3:1. After cooling down to room temperature naturally, the ball mill tank was unscrewed in a nitrogen-filled glove box to collect BP and grind it for later use.

### 2.3. Preparation of PEEK/PTFE/BP Composite

Firstly, the powder mixtures of PEEK, PTFE, and BP were put into the ethanol (liquid-solid mass ratio: 1:1), and the mixed solution was stirred for 0.5 h at room temperature. After that, the mixed solution was poured into a ball mill tank and mechanically ground at a speed of 300 rpm for 2 h. Then, the mixed powder was placed into a vacuum drying oven at a temperature of 70 °C for 6 h. Afterwards, it was ground and filtered through an 800-mesh screen.

**Figure 2:**
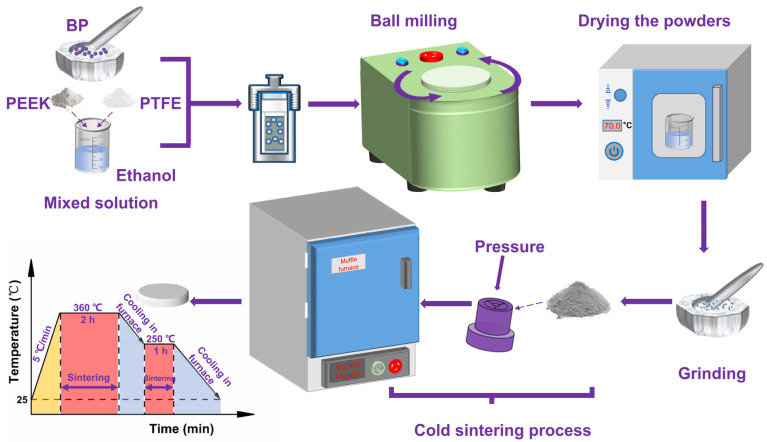
The schematic diagram of the preparation of PEEK/PTFE/BP composites through the cold sintering process.

The mixed powder was pressed under a pressure of 100 MPa and sintered in a vacuum muffle furnace at 360 °C. The sintering procedure is shown in Figure 2. Finally, the prepared composite materials were pure PEEK (P1), PEEK/10 wt% PTFE (P2), PEEK/10 wt% PTFE/0.5 wt% BP (P3).

### 2.4. Frictional Tests and Characterizations

#### 2.4.1. Characterizations of Red Phosphorous (RP) and Black Phosphorous (BP) Nanosheets

The use of X-ray diffraction (D/Max 2550, Rigaku, Akishima, Japan) and Raman spectra (LabRAM HR Evolution, Horiba, Japan) with a laser of 532 nm and a power of 50 mW were adopted for the characterization of the RP and the prepared BP. The high resolution transmission electron microscope (HR-TEM, FEI Tecnai G2-F20, Hillsboro, OR, USA) was used to examine the morphology of the BP. Atomic force microscopy (AFM, Bruker Dimension ICON, Santa Barbara, CA, USA) was used to determine the thickness of the BP.

#### 2.4.2. Frictional Tests and Characterizations of PEEK Composite Materials

The tribotests of the composites were conducted with a universal mechanical tester (UMT-5, CETR, Campbell, CA, USA) with a ball-on-disk configuration. GCr15 bearing steel balls with a diameter of 4.68 mm and a surface roughness *S_a_* of 50 nm were chosen as the tribopair materials, and they were rinsed with alcohol prior to each experiment. Tests were performed under normal loads of 3 N at a frequency of 5 Hz, and the reciprocating friction stroke was 5.0 mm.

The morphologies of worn tribopair surfaces were investigated by using the scanning electron microscopy (ZEISS, Jena, Germany, GeminiSEM 300, Signal A = SE2, Vac < 10^−3^ Pa, beam current = 72.9 μA) and energy dispersive X-ray spectroscopy (Oxford Xplore30, Oxford, UK). The wear volumes of the worn composite were measured using a 3D white light interference surface topography device (Nexview NX2, Zygo, Middlefield, CT, USA). The static contact angles of the composites were measured with 50 μL distilled water, normal saline (AS-ONE, Osaka, Japan) and calf serum (Pingrui Biotechnology, Beijing, China) by using a contact angle instrument (OCA-25, DataPhysics, Filderstadt, Germany).

### 2.5. Antibacterial Experiment by Film Sticking Method

**Figure 3:**
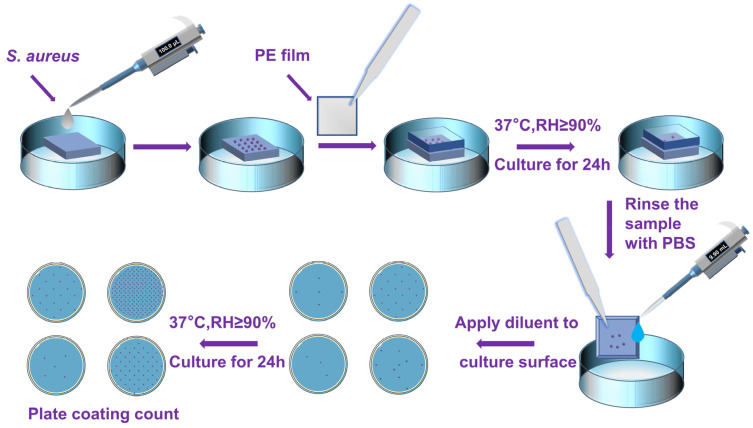
The schematic diagram of the antibacterial experiment process.

The sample of PEEK/10 wt% PTFE, PEEK/10 wt% PTFE/0.5 wt% BP, and polypropylene covering film were sterilized in alcohol for 30 min and dried to reserve. After dropping 100 µL *S. aureus* suspension cultivated by LB medium with a concentration of 10^6^ CFU/mL to the sample (size: 20 mm × 20 mm × 2 mm), the sample was covered with PP film using sterile forceps to ensure that the bacteria contacted the sample evenly, and then kept in a 37 °C incubator (SLI-1200, Sanyo, Osaka, Japan) for 24 h. The sample and the covering film were washed with 9.9 mL sterile PBS solution (diluted 100 times), and the collected solution was continuously diluted 10 times with the PBS solution. Then, the appropriate dilution ratio of 100 μL bacteria solution was applied to the surface of the medium and kept in a 37 °C incubator for 24 h. After culture, photos were taken in order to count the colony values of all the plates. The antibacterial experiments (the procedure as described in Figure 3) were repeated 3 times to reduce the experimental error. According to HG/T 3950-2007, the bacterial inhibition rate (%) was represented in Equation (1):

R (%) = (C − E)/E × 100%(1)

R: Antibacterial rate, %(2)

C: Concentration of control group, CFU/mL(3)

E: Concentration of experimental group, CFU/mL(4)

C(E): CFU × dilution ratio × 10/mL, CFU/mL(5)

## 3. Results and Discussion

### 3.1. Characterizations of RP and BP

**Figure 4:**
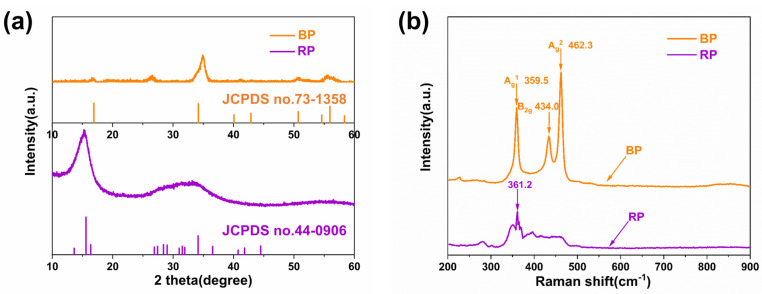

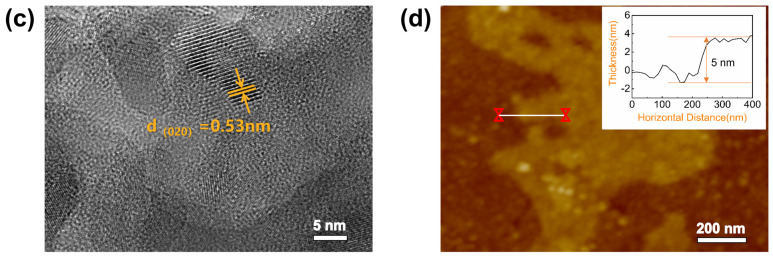
The Raman spectra (**a**) and X-ray diffraction (**b**) of RP and BP nanosheets; the HR-TEM image (**c**) and AFM image (**d**) of the BP nanosheets.

According to the XRD analysis of Figure 4a, the XRD peaks of BP prepared by high-energy ball milling were consistent with those of pure BP (JCPDS no.73-1358), with sharp diffraction peaks and good crystallinity. Raman spectra confirmed the information about the BP, as shown in Figure 4b. The characteristic peaks of BP appeared at 359.5 cm^−1^, 434.0 cm^−1^, and 462.3 cm^−1^, corresponding to three atomic vibration modes A_g_^1^, B_2g_, and A_g_^2^ of the phosphorus atom, respectively [2]. Software digital micrograph analysis of the HR-TEM image in Figure 4c showed that the lattice spacing of the prepared BP nanosheet was 0.53 nm, which was attributed to d-spacing of (020) crystal lattices as reported in the literature [32,47]. The lamella thickness of the BP nanosheet (see Figure 4d) was 5 nm, which proved the ultra-thin morphological characteristics of the prepared nanosheets.

### 3.2. Tribological Properties and Analysis of Composite Materials

**Figure 5:**
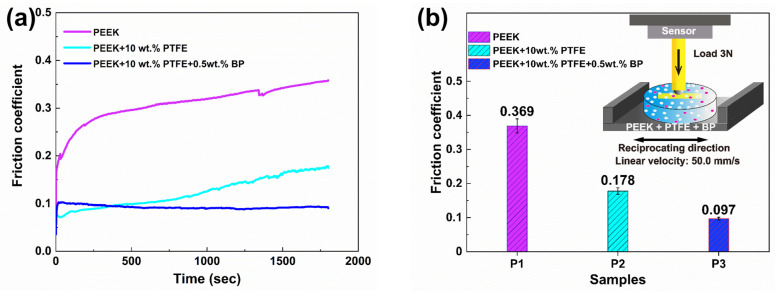

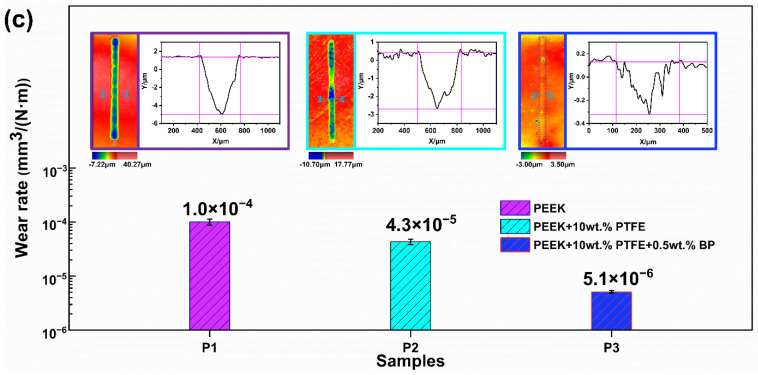
The friction coefficients and wear rates of the samples. (**a**) Variation curves in the COFs as a function of time for three samples; (**b**) The average COFs of three samples; (**c**) The wear rates of three samples.

The coefficients of friction (COFs) of the samples under 3 N at the sliding speed of 50.0 mm/s were summarized and shown in Figure 5. The COF of pure PEEK was relatively low at first, and then it gradually increased to 0.369. After the incorporation of 10 wt% PTFE, the COF reduced from 0.369 to 0.178. After 0.5 wt% BP nanosheets were added into the PEEK/PTFE composite, the lubrication performance of the composite was greatly improved, and the COF reduced significantly, with a minimum COF of 0.097. Figure 5c presented the wear rates of three samples P1, P2, and P3. The wear marks of the polymers were summarized by using a three-dimensional interference surface topography device, and the wear rates were calculated using ZYGO MetroProX software. Compared with pure PEEK, the wear rate of the composite after the addition of 10 wt% PTFE filler obviously decreased from 1.0 × 10^−4^ mm^3^/(N m) to 4.3 × 10^−5^ mm^3^/(N m). Similarly, the incorporation of BP significantly reduced the wear rate of the composite to 5.1 × 10^−6^ mm^3^/(N m). On the whole, compared with pure PEEK, the COF reduced by about 73%, and the wear rate decreased by approximately 95% for PEEK/10 wt% PTFE/0.5 wt% BP.

**Figure 6:**
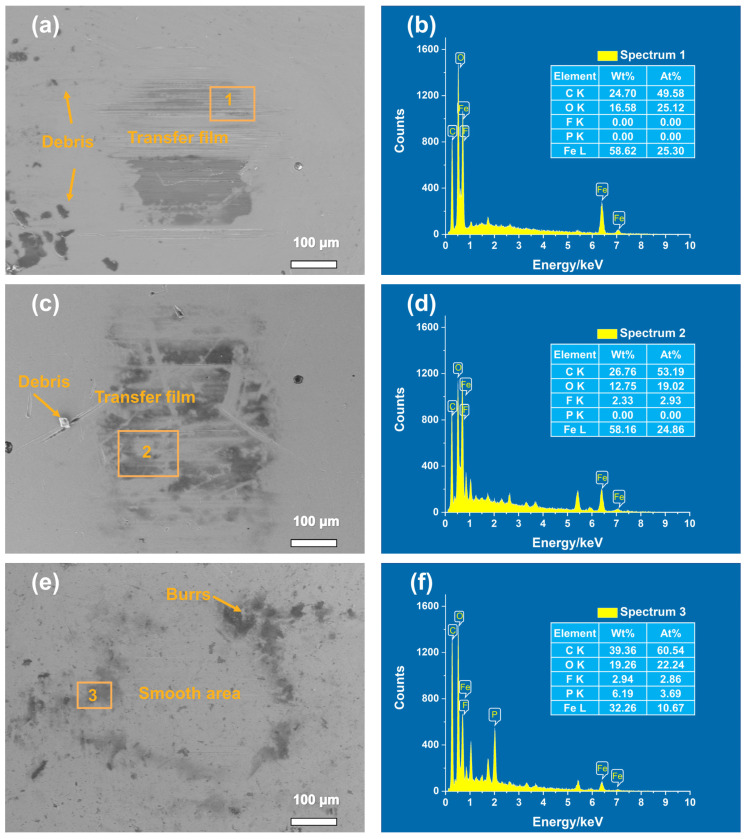
SEM-EDX micrographs of the bearing steel ball surfaces sliding against different composites: (**a**) pure PEEK; (**b**) EDX image of area 1; (**c**) PEEK/10 wt% PTFE; (**d**) EDX image of area 2; (**e**) PEEK/10 wt% PTFE/0.5 wt% BP; (**f**) EDX image of area 3. (EHT = 10.00 kV, Mag = 160×).

The SEM-EDX micrographs of the bearing steel ball surfaces after the dry friction condition were presented in Figure 6. As can be seen from Figure 6a, the thick transfer film formed on the spherical surface of the PEEK had so poor wear resistance that it easily fell off and produced fragments during the reciprocating motion. The double formation of PEEK and PTFE mixed transfer film (see Figure 6b) significantly reduced the COF. The transfer film (see Figure 6c) formed on the spherical surface of the PEEK/PTFE/BP composite was relatively smooth, thin, and uniform. The transfer film contained higher phosphorus (P) and oxygen (O) elements (see Figure 6f), which proved that BP can form a stable transfer film at the sliding interface. Because the BP layers were combined by van der Waals force, the BP transfer film had good adhesion with the tribopairs, which can protect the tribopairs from wear.

**Figure 7:**
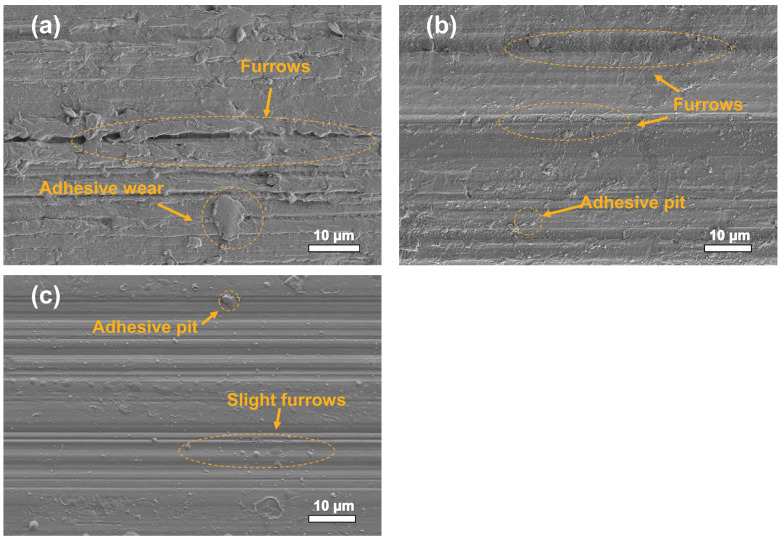
The SEM micrographs of the worn surfaces of different composites: (**a**) pure PEEK; (**b**) PEEK/10 wt% PTFE; (**c**) PEEK/10 wt% PTFE/0.5 wt% BP. (EHT = 3.00 kV, Mag = 1600×).

The SEM micrographs of the worn surfaces of different composites were shown in Figure 7. Rough furrows were parallelly distributed on the PEEK worn surface (see Figure 7a), showing severe adhesive wear. This was due to the dimensional cutting of the PEEK materials by the micro convex body on the surface of the steel ball, resulting in the plastic deformation and furrow effect of PEEK. The addition of PTFE (see Figure 7b) reduced the mechanical wear to a certain extent. However, the worn surface of the PEEK/PTFE/BP (see Figure 7c) was relatively smooth with very slight furrows. Therefore, BP reduced the COF and improved the wear resistance of the PEEK composites.

### 3.3. Biological Wettability and Antibacterial Property

**Figure 8:**
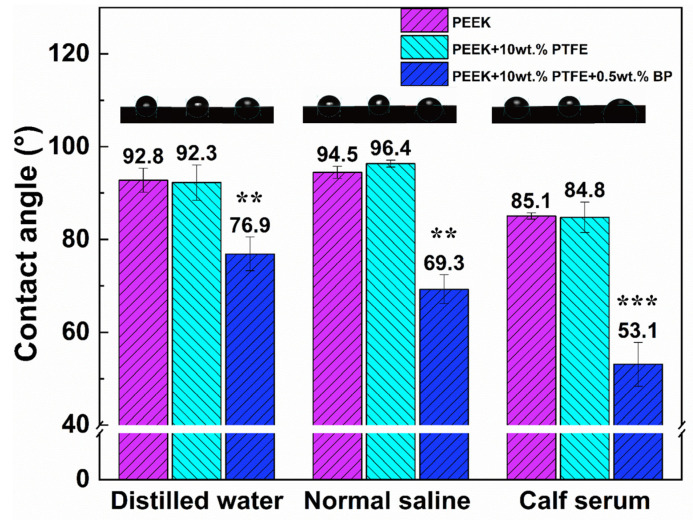
The contact angles of the composites with distilled water, normal saline, and calf serum. (**: *p* < 0.01, ***: *p* < 0.001, statistically significant difference; PEEK vs. PEEK/PTFE, PEEK/PTFE/BP).

The static contact angle values of different composites under distilled water, normal saline, and calf serum were shown in Figure 8. It can be clearly seen that there was no significant difference between the contact angle values of pure PEEK and PEEK/10 wt% PTFE for the three liquids mentioned. The PEEK/10 wt% PTFE/0.5 wt% BP showed the optimum wettability with contact angle values of 76.9°, 69.3°, and 53.1° under distilled water, normal saline, and calf serum, respectively. The addition of BP could diminish the surface tension of the liquid and improve the biological wettability of PEEK composites, which might reduce the biological responses for the implant materials.

**Figure 9:**
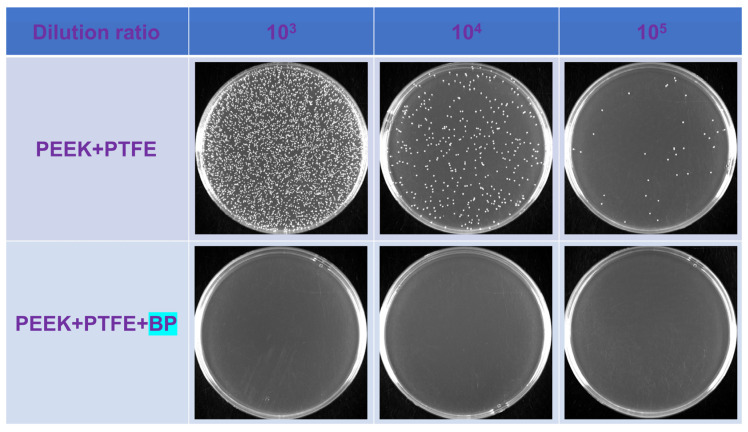
Photographs of agar plates of *S. aureus* after incubation with PEEK/10 wt% PTFE and PEEK/10 wt% PTFE/0.5 wt% BP.

The photographs of the agar plates of *S. aureus* after incubation with PEEK/10 wt% PTFE and PEEK/10 wt% PTFE/0.5 wt% BP were presented in Figure 9. The group of composites added to BP can effectively inhibit the reproduction of *S. aureus*. The antibacterial rates of PEEK/PTFE/BP calculated by three repeated experiments were 99.9% (see Table 1), which were evaluated as class I strong antibacterial material based on HG/T 3950-2007. Many studies on BP nanosheets as antibacterial materials have been conducted [48,49,50,51], indicating that the surface of BP nanosheets could produce reactive oxygen species, destroy bacterial cell membranes, and inhibit bacterial reproduction.

## 4. Conclusions

In summary, this research investigated the tribological behavior and wear mechanism of PEEK/PTFE with the addition of BP, and conducted biological wettability and the antibacterial experiments. Compared with pure PEEK, the COF of PEEK/10 wt% PTFE/0.5 wt% BP was reduced by about 73% (from 0.369 to 0.097) and the wear rate decreased by approximately 95% (from 1.0 × 10^−4^ mm^3^/(N m) to 5.1 × 10^−6^ mm^3^/(N m)) owing to the lubrication of the BP transfer film, making PEEK composite materials more wear-resisting for use in artificial joint implants.

In addition, BP endowed the PEEK composites with excellent biological wettability and antibacterial properties. It was measured that PEEK/PTFE/BP was considered as class I antibacterial material owing to its antibacterial rate above 99.9%, which was helpful to solve the problem of adverse infection reaction caused by PEEK materials implanted in the body.

PEEK/PTFE/BP composites can realize blending and granulation, and are suitable for 3D printing and injection molding. Thus, it is expected that the research results will provide a potential opportunity for an extensive range of applications for PEEK artificial joint materials. The formulation is prior to commercial PEEK production, which still requires improved mechanical properties and extensive clinical biological tests.

## Figures and Tables

**Table 1:** A summary of the antibacterial rates of three repeated antibacterial experiments.

Serial Number	Concentration of the Colon (CFU/mL)		Antibacterial Rate (%)
	Control Group	Experimental Group	
1	3.4 × 10^8^	<10^4^	99.9
2	5.8 × 10^9^	<10^4^	99.9
3	1.5 × 10^9^	<10^4^	99.9

## Data Availability

Not applicable.
